# Evaluation of Internet-Based Technology for Supporting Self-Care: Problems Encountered by Patients and Caregivers When Using Self-Care Applications

**DOI:** 10.2196/jmir.957

**Published:** 2008-05-15

**Authors:** Nicol Nijland, Julia van Gemert-Pijnen, Henk Boer, Michaël F Steehouder, Erwin R Seydel

**Affiliations:** ^2^Department of Technical and Professional CommunicationFaculty of Behavioural SciencesUniversity of TwenteEnschedeThe Netherlands; ^1^Department of Psychology and Communication of Health and RiskFaculty of Behavioural SciencesUniversity of TwenteEnschedeThe Netherlands

**Keywords:** Internet, technology, primary care, access to information, electronic patient-provider communication, self-care

## Abstract

**Background:**

Prior studies have shown that many patients are interested in Internet-based technology that enables them to control their own care. As a result, innovative eHealth services are evolving rapidly, including self-assessment tools and secure patient-caregiver email communication. It is interesting to explore how these technologies can be used for supporting self-care.

**Objective:**

The aim of this study was to determine user-centered criteria for successful application of Internet-based technology used in primary care for supporting self-care.

**Methods:**

We conducted scenario-based tests combined with in-depth interviews among 14 caregivers and 14 patients/consumers to describe the use of various self-care applications and the accompanying user problems. We focused on the user-friendliness of the applications, the quality of care provided by the applications, and the implementation of the applications in practice.

**Results:**

Problems with the user-friendliness of the self-care applications concerned inadequate navigation structures and search options and lack of feedback features. Patients want to retrieve health information with as little effort as possible; however, the navigation and search functionalities of the applications appeared incapable of handling patients’ health complaints efficiently. Among caregivers, the lack of feedback and documentation possibilities caused inconvenience. Caregivers wanted to know how patients acted on their advice, but the applications did not offer an adequate feedback feature. Quality of care problems were mainly related to insufficient tailoring of information to patients’ needs and to efficiency problems. Patients expected personalized advice to control their state of health, but the applications failed to deliver this. Language (semantics) also appeared as an obstacle to providing appropriate and useful self-care advice. Caregivers doubted the reliability of the computer-generated information and the efficiency and effectiveness of secure email consultation. Legal or ethical issues with respect to possible misuse of email consultation also caused concerns. Implementation problems were mainly experienced by caregivers due to unclear policy on email consultation and the lack of training for email consultations.

**Conclusions:**

Patients’ and caregivers’ expectations did not correspond with their experiences of the use of the Internet-based applications for self-care. Patients thought that the applications would support them in solving their health problems. Caregivers were more reserved about the applications because of medico-legal concerns about misuse. However, the applications failed to support self-care because eHealth is more than just a technological intervention. The design of the applications should include a way of thinking about how to deliver health care with the aid of technology. The most powerful application for self-care was secure email consultation, combined with a suitable triage mechanism to empower patients’ self-awareness. Future research should focus on the effectiveness of such Web-based triage mechanisms for medical complaints and on the development of interactive features to enhance patients’ self-care.

## Introduction

Internet-based technology has become increasingly important for promoting access to care and self-care management [1-3]. Particularly, systems that combine high-quality information with interactive components for self-assessment, decision support, or behavior change have the potential to reduce costs while maintaining the same or achieving better quality of care [2,4]. This means that technology can respond to an increasing demand for care in the aging society.

What has become widely accepted is the value of Internet-based technology to deliver health care irrespective of time and place, and the enhanced access to care for people from underserved areas [1,3]. Notwithstanding the better services, a relevant question is whether these Internet-based applications can support patients or consumers in controlling their own health behavior, and secondly, whether they can facilitate the quality of health care.

Recognizing that patients are interested in managing their own health, the industry is exploring ways of encouraging them to be more in control of their own health and health care [5]. Initially, health care innovations were mainly market-driven products delivering information that may not benefit patients. Currently, innovative Web-based technologies in health care that have interactive components, such as an “ask the doctor service” (via secure email consultation) [1] and self-tests, are evolving rapidly [6]. The use of the Internet is no longer restricted to information retrieval but enables patients to manage their own health proficiently and at their own convenience by means of such interactive components for self-care.

When self-care is the focus of Internet-based technology, we need to evaluate more thoroughly what people can do with the self-care applications. How do they evaluate their own health condition with self-assessment tools, what do they feel and think while communicating with a system about their ailment, and what do they expect from computer-generated self-care advice? A qualitative evaluation study is thus needed to achieve insight into the process of consulting Internet-based applications for medical support and to determine which health care functions can be delegated to Internet-based health care systems [2].

To date, evaluations that take user perspectives into account as well as the appropriateness and meaningfulness of interactive components to support self-care are scarce [2,7]. The aim of this study was to determine user-centered criteria for successful application of Internet-based technology for supporting self-care. To this end, we evaluated the use of three Internet-based applications in primary care that have various features for self-care (eg, self-test, digital triage) and electronic patient-caregiver communication (free text or question-and-answer form).

In wanting to observe the contribution of various interactive components to support self-care, we focused on the user-friendliness of the applications [2,3,8,9], the quality of care provided by the applications [2,10], and the implementation of the applications in practice [11].

## Methods

### Description of Internet-Based Applications for Self-Care

We evaluated three commonly used Internet-based primary care applications in the Netherlands: Medicinfo (M) [12], Praktijkinfo (P) [13], and Dokterdokter (D) [14]. These certified applications are based on ISO 9000:2000 standards [15] and use encrypted software for secure exchange of information. Users have to log on with a user ID and password. Patients have free access to all three applications.

The applications have multiple components for self-care so as to appeal to a wide range of users, thus underlining that patients will differ in their needs for self-care. In all three applications, patients can search for self-care information about their health complaint by means of a digital medical encyclopedia with alphabetically ordered lists or online health brochures. Two applications, M and D, provide self-care tools that can be used for various purposes: obtaining information about the possible causes of a health complaint, and checking the necessity of a doctor’s visit and getting (self-care) advice for nonurgent health complaints.

For the first purpose, application M provides a so-called Symptom Scan. This self-test consists of a questionnaire about specific health symptoms and generates a bar chart showing the probabilities of medical causes for a certain disease or injury.

For the second purpose, M and D provide a digital triage function that consists of a symptom-driven question-and-answer system for filtering urgent complaints and for providing fully automated diagnosis and advice. The digital triage is intended to prevent unnecessary visits to the doctor. Patients have to label their health complaint either on alphabetically ordered lists (M) or on a virtual body (D). Subsequently, they have to run through the questions and answers related to the identified problem. In the event of urgent symptoms, the triage application generates advice to visit a doctor. In the event of nonurgent issues, it generates tailored self-care advice.

All three applications offer the possibility of secure email communication between patient and caregiver. The P and D applications provide online encounters between patient and general practitioner (GP) but require a pre-existing relationship. Patients of M can consult 28 specific health experts anonymously. With M and P, patients can consult a caregiver in their own words (free text). With D, patients first have to run through a question-and-answer system (digital triage) before being able to pose their question in their own words. Questions have to be answered within 24 hours, and caregivers receive a reimbursement for each Web consultation.

### Recruitment of Participants

Fourteen caregivers participated in this study, including GPs, physicians specializing in communicable diseases, and a psychologist. All caregivers were current users of one of the Internet-based care applications. Participating caregivers were recruited by email by the systems’ providers and used their practice website and email to recruit patients. A total of 14 patients agreed to participate. Eligible patients were at least 18 years old, Dutch speaking, and had experience with using one of the Internet-based applications.

### Scenario-Based Tests Combined With In-Depth Interviews

We used scenario-based tests combined with in-depth interviews to describe the use of the Internet-based applications and the accompanying user problems. Trained observers watched users communicating with the interface of the application while doing simulated tasks and thinking aloud [16]. The test consisted of six “what if” scenarios (see Multimedia Appendix) representing health complaints related to self-limiting diseases. All scenarios were tested by physicians. Patients were instructed to read a scenario out loud and to imagine that they were in the situation described. Caregivers, on the other hand, were instructed to answer patients’ questions. The participants’ activities were recorded with audio-visual equipment. The tests were carried out at the participants’ home or workplace. Each test lasted about 90 minutes.

### Data Analyses

Two researchers independently identified user problems from the verbal reports of the scenario-based tests. Repeated or reworded descriptions of the same problem were only counted once. Agreement on categorization of the problems was high [17], both for the patient problems (Cohen’s kappa = 0.95) and the caregiver problems (Cohen’s kappa = 0.87). In the event of disagreement, researchers discussed the categorization of the problems in order to reach consensus. All of the 358 identified user problems were categorized as quality demands for supporting self-care by technology [10]:

1. Problems with user-friendliness: referring to technical and design features (presentation of information) that are relevant to the use of the applications
2. Problems with the quality of care: referring to patient-caregiver communication and self-care advice generated by the application, especially the responsiveness of the applications [18,19]
3. Implementation problems: referring to the incorporation of the applications into daily practice and to policy issues concerning email consultation

## Results

The results present the problems observed while using the applications for self-care aims. The results section is split into two parts: the first addresses patients’ use of the applications and the problems experienced, and the second addresses caregivers’ use of the applications and the problems experienced with handling patient requests. To indicate the main problems, a full overview is given for each.

### Patient Problems

#### Searching for Self-Care Information

By means of digital medical encyclopedia with alphabetically ordered lists of medical terms, patients could seek self-help information about their health complaint. Patients experienced difficulties in finding information. The navigation structure of the website (home page) appeared troublesome for patients trying to find the information they were looking for. For instance, the search options were not equipped for finding the right information quickly and also provided irrelevant or useless results. As patients wanted to retrieve health information with as little effort as possible, and the applications did not meet this need, they opted for a search engine, such as Google, to find the right information.

P13 Because I can’t find a “search function” and the structure of the menu is unclear, it means that I have to carry on scrolling. For me, that’s a big enough reason for quitting this site. It’s just too much bother, and I’m someone who uses the Internet on a daily basis.

P8 With Google, you get the right answer straight away. It’s much faster than this. I can’t ask my question here. I have to search.

Semantic shortcomings hindered the search process because the search options used medical terms that were not defined or explained, which meant that patients could not match their health complaint with the terminology offered.

P10 I read “muscular weakness.” Now what is muscular weakness?

P3 Lots of difficult words. Better information about what it is would be handy.

Comprehension problems arose because the virtual body of the application did not provide sufficient information for labelling a health complaint. Patients had to click on the body to label their complaint in order to get more information. However, patients were not accustomed to describing their complaint via the labels of a virtual body, and they were not able to label ailments like tiredness, insomnia, and mental problems. The possibilities offered by the medical encyclopedia were often irrelevant and/or too general to be helpful for self-care.

P7 I expect the ABC [medical encyclopedia] to comprise both physical and mental problems. I am now looking for sleep disorders, but that isn’t my main problem. Apparently I first have to make a diagnosis about what’s wrong with me before I can search further.

P5 I was expecting more of a medication advice. This information just deals with common solutions. I find that general knowledge.

#### Interpreting Computer-Generated Self-Care Advice

Via self-tests and digital triage features, patients could receive fully automated self-care advice to identify the possible causes of a health complaint or to decide whether a doctor’s visit was necessary.

M provides a so-called Symptom Scan, a self-test to gather information on the possible causes of a health complaint. The self-test can be consulted for four health complaints: dizziness, chest pain, headache, and tiredness. It consists of a list of questions about specific symptoms. The self-test generates a list of probabilities of medical causes for a certain disease or injury; for example, a test for headache resulted in a 96% chance of migraine, a 1.1% chance of a brain tumour, and a 0.1% chance of meningitis.

Patients had difficulty interpreting the results of the Symptom Scan. It was unclear to them how they should interpret a percentage of 0.1. Is this chance negligible or is it a realistic 0.1% chance of meningitis? As the system failed to provide further information on this, a doctor still needed to be consulted. The system thus did not provide the security the patient was seeking or support the patient in his or her self-care demand. In certain cases, the test results even evoked fear. This was due to the fact that most of the presented terms were related to injuries and diseases instead of common conditions. Furthermore, patients noted that in many cases the questions of the self-test were irrelevant or incomplete. The consequence of this was that patients lost confidence in the Symptom Scan and no longer took the results of the test seriously. Besides this, the patients appeared to have insufficient expertise to answer the Symptom Scan’s questions; consequently, the results did not coincide with the patient’s complaint.

P6 It doesn’t help me much. A percentage of 0.3—I have no idea what that means. In my opinion, those questions were totally irrelevant.

Patients could check the necessity of a visit to the doctor by means of a symptom-driven question-and-answer system (digital triage). Patients felt that they were referred to a doctor too quickly. Consequently, the advice to visit a doctor was not always taken seriously, particularly in the case of an apparently less serious health complaint, like a cough. Moreover, the generated advice frightened patients when they were told to visit a doctor after answering only a few questions.

P8 Sounds ominous: “Contact your GP.” I would prefer some explanation why that is necessary.

What do patients expect from computer-generated self-care advice? The question-and answering system (digital triage) seemed appealing to patients because of its ability to adjust to personal characteristics (ie, patients fill in their personal symptoms and the system responds to their personal data). The fact that patients have to fill in personal information results in an expectation of tailored health care advice. However, patients found the self-care advice to be insufficiently tailored to their specific needs; it was no different from the general information available in public health leaflets or encyclopedia. Consequently, patients attached greater importance to personal advice from a caregiver, whether through the Internet or from a doctor’s visit.

P11 I am quite interested in what it comes up with, whether it’s identical to what has been said before [in the medical encyclopedia] or if I will be given more specific information on my current symptoms.

Furthermore, patients found that the digital triage function did not yield as much as expected. The number of questions they had to answer on an ailment was not in accordance with the perceived severity of their health problem. For example, for a problem like a cough, patients had to answer about 50 questions before they received advice on what to do (application D). Patients found the number of questions disproportionate to their complaint. With more a complex health problem, such as tiredness, patients had fewer objections to a greater number of questions because they understood that more questions are needed if a complex problem is to be considered.

P2 That cough question, it takes you 15 minutes to run through all the questions, whereas you might just as well have picked up the telephone.

#### Formulating Health Complaints via Email

Patients faced problems describing their health problem; mental health problems were especially difficult to verbalize. In these cases, patients were already heading for a doctor’s visit during their email consultation. One of the applications (P) requires patients to classify their complaint under a category such as shoulder complaint or headache before they can pose a question to their GP by email. These rubrics appeared insufficiently tailored to the language patients used for verbalizing their complaint.

P13 It’s quite tricky, having to categorize your question. Look, if you have cystitis, it’s not so difficult. But if you think you’ve got a pain in your stomach, or are constipated, those kinds of things are difficult to classify.

Patients also found it difficult to decide what kind of information a caregiver needs in order to be able to answer their questions. The completeness of information given to a caregiver depended on the type of interaction with him or her. In the event of a pre-existing relationship, patients anticipated the GP’s knowledge about their medical history (information about their personal situation and activities that had already been undertaken to solve the health problem). When consulting an unknown caregiver, patients gave as much information as possible about their personal situation and health problem, often accompanied with information about the actions they already had undertaken. By doing so, patients took into account the fact that the caregiver could not pose a counterquestion because of the lack of feedback features. With application M, patients can consult several clinical experts for advice on a specific health problem; however, it appeared to be difficult for patients to choose the right expert for their complaints (eg, they found it difficult to select an expert for a complaint of headache).

#### Implementation of Applications in Practice

Patients were not trained to use the self-care applications. Moreover, they had no idea whether use of the applications would continue to be free in the future. Due to lack of training or education, not all features of the applications were used, such as the possibility for patients to store the information generated by the applications (P and D) in a patient file. The structure of the websites seemed so unclear that all kinds of features to document and upload information were overlooked.

#### Overview of Patient Problems

Table 1 presents an overview of the problems patients experienced while they were observed using the applications’ features for controlling their health. Problems were categorized into quality demands for supporting health care through technology. Patients experienced 260 problems in total. They faced problems mainly with the quality of care provided via the Internet-based applications. The information was insufficiently tailored to patients’ needs, and language (semantics) appeared one of the main obstacles to providing appropriate and useful self-care advice. Problems with the user-friendliness of the applications were mainly related to navigation features, such as inadequate search options and unclear presentation of information; the menu on home pages failed to enable patients to find the information they were looking for. Implementation problems occurred because of vagueness concerning regulations about free access and lack of training on how to use the applications for solving health-related problems.

**Table 1:** Overview of patient problems (N = 260)

Quality Demand	Identified Patient Problems
User-friendliness (n = 106, 40.8%)	Navigation problems: Lack of a search engine Lack of an adequate search option Unclear navigation structure; hyperlinks were nonexistent or useless Unclear or unattractive layout of Web pages No features for printing information
	Technical problems: Software bugs Drop-down menus or back buttons failed
Quality of care (relevance, comprehensibility of information; responsiveness) (n = 146, 56.1%)	Problems with relevance of information: Information provided by the digital medical encyclopedia was too general to be useful Information provided by the virtual body was too limited to be useful Self-care advice insufficiently tailored to personal needs
	Problems with comprehensibility of information: Semantic mismatch between system and users because of unclear medical terms and lack of features to verbalize a problem in their own vocabulary Self-care advice hard to interpret Self-care advice frightening
	Problems with responsiveness: Caregiver used more than prescribed response time to answer patients’ questions
Implementation (policy, training) (n = 8, 3.1%)	Lack of education: Underuse or misuse of applications because of lack of education Uncertainty about regulations for using Internet for self-care

### Caregiver Problems

#### Identification of Patients

In the event of a pre-existing relationship between a patient and caregiver, the caregiver first looks up the name and date of birth of the patient in order to identify him or her. Next, the caregiver looks for additional information in his or her own patient record. Although caregivers authenticate the patients by checking the personal data, they still have concerns about the service being misused (ie, they might receive requests from unknown patients who were using the account of a patient already on file). In case of anonymous email encounters, caregivers were also aware of the risk of not knowing the patient. With application M, they are trying to curtail this by asking all patients approaching them for an email consultation to fill in a health statement first. To this end, patients must answer questions specifically selected with regard to what the caregiver needs to know as well as the health risks the patient might run. In this way, the caregiver can soon see in an overview how or where he or she must adjust the advice to the situation of the unknown patient. All the questions have to be answered with “No” if a patient desires an email consultation. The health statement does not eradicate all risk, however.

C7 Because that’s the last thing you want, right? That they leave with wrong advice but then it turns out that we did ask the question only that they didn’t answer it, that they thought, “Oh, it’s not a problem,” which later turns out to be one after all. That’s the drawback of not knowing somebody and still advising them on the basis of a health statement that they have had to fill in themselves.

#### Interpreting Patient Requests

For the P and D applications, email communication is only possible with registered patients. In this way, it is clear to the caregiver who is asking the question. For M, the people asking the questions are anonymous, which means the caregiver has no background information on the person concerned. However, to be able to give a more personal or tailored answer, it is necessary to have background information or a medical history.

C7 It can be difficult sometimes. You only have a smidgen of background information about somebody, whereas with real-life contact you can see how someone reacts. When you say something and the message does not come across at all, someone starts to look vague or something, then you can try to explain it again in a different manner, but this way you just don’t see anything, so it’s difficult. If someone hardly gives background information, you have to keep your advice rather general, but when somebody imparts a good deal of background information, your answer can be more exhaustive.

With application D, caregivers received a history of the patient’s health problem via the questions and answers from the digital triage system. Although the caregivers valued the medical history questionnaire differently, they remarked that it offered many advantages when interpreting the patient request. In their opinion, it offered a lot of information that helped to understand the complaint or the problem better and thus allowed them to distinguish important alarm signals. On the other hand, the medical history questionnaire appeared insufficiently capable of analyzing the health complaint to result in clear advice. It took too long to filter the relevant information.

C6 Look, if all I can see is “No” everywhere [answer indicating nonurgent symptoms], I am inclined to stop reading all the answers and overlook the “Yes.”

#### Answering Patient Requests

Aware that their written answers can have legal consequences, caregivers take great care with the formulation of their answers to patients. Moreover, with the absence of a clear protocol for communicating online with patients, caregivers also worry about the quality of care. With application M, caregivers are alert to mentioning that their advice could be a possible indication of the cause of the complaint, but that it is not a diagnosis.

C5 Well, I’m always on my qui vive, so as not to write things down in the file that could later be used against me in court, shall we say. So I tread cautiously with the formulation of a number of things.

C9 You can give general advice. You can always do that, but you have to incorporate a kind of safety device by saying “Oh, in a number of cases, there will be exceptions.” And that’s why we are constantly pleading for a quality protocol for these kinds of things, and that protocol must comprise three elements: expertise of the person manning the desk—it must be someone with considerable experience; there must be a certain guarantee that the questions will be answered within a certain time limit; and the third, and that is the trickiest of them all, is that you must try to give answers that are safe, and...if you think “There’s a risk here,” you must also clearly communicate that with...“If you want to be sure, you must make an appointment.”

With application D, the digital triage generated a standard advice (ready-made answer) based on an ICPC code. In the Netherlands, the International Classification of Primary Care (ICPC) is accepted as a standard for coding and classifying health complaints, symptoms, and health disorders in primary care [20]. In most cases, the generated ICPC code did not correspond with the caregiver’s expectations. Sometimes an ICPC code could not even be generated and the caregivers themselves had to allocate a code, which was not always easy due to lack of relevant medical information. Moreover, the ready-made answers did not correspond with the professional beliefs of practising medicine and, as a consequence, they were changed or reformulated (ie, geared more to the personal and/or medical characteristics of the patient).

C8 It’s just too general. I have to rewrite things quite often. And not all questions from patients refer to an illness. I remember someone asking me once about genetic research. That’s not a medical problem. Things are not always run-of-the-mill.

#### Documentation of Patient Requests

The system’s features, like sending attachments and archiving patients’ questions and answers, were hardly used due to a lack of education about the usage of the applications. Furthermore, despite most caregivers wanting to know how patients acted on their advice, two of the applications (M and D) did not offer a feedback feature. Caregivers thus emphatically advised patients to visit a caregiver in case of doubt about their health problem.

C7 I find it quite difficult at times, when I get so little feedback on how my answer has been interpreted. Was it successful or not?

C9 It’s true it’s difficult, because you’re not given any feedback. If the patient doesn’t react, fine, but if that leads to mistakes being made, that’s a pitfall.

The medical records of caregivers’ patients could not be integrated with the documentation system of the Internet-based applications. Although patients’ demographics and medical histories could be saved, caregivers did not use this functionality because they found it inconvenient. All notes on an email consultation, including date and content, were made in their own medical records.

C11 At this moment I still don’t have the option to look at information coupled to my medical record. And no link to your own record is inconvenient.

C13 If something really special has to be recorded, then I would do so in my medical record. I regard this [application P] merely as a means of communication, whereby I do not feel the need to document patient information.

#### Implementation in Practice

Caregivers faced difficulties with the incorporation of email consultation into daily practice. The Internet-based care applications were not compatible with the patient administration systems already in use, and email consultation usually takes place outside of office hours. Moreover, caregivers were ignorant about the conditions (rights and obligations) of email consultation. Directives for the use of electronic patient-caregiver communication were unavailable or unclear about the care delivery process and the definition of a pre-existing relationship. Caregivers wondered whether a personal encounter was required before an online encounter and about the definition of the first personal contact. Moreover, they expected greater inspection from government on the influence of health care insurers regarding privacy. They also felt the need for an unambiguous view on the admission of email communication for anonymous contact between patient and caregiver. Caregivers are of the opinion that the rate of a Web consultation (€4.50) is too low. They think that although email consultation can be an added value to regular care because access to care could be enhanced, they would restrict its use to simple nonurgent health complaints and to known patients.

#### Overview of Caregiver Problems

Table 2 presents an overview of problems faced by caregivers while using the applications for handling patient requests. Caregivers experienced 198 problems in total. About half of the problems concerned the user-friendliness of the applications, such as unclear navigation structures and lack of feedback or documentation possibilities. Quality of care problems concerned laborious answer procedures, the nonprofitability of email consultation, and legal or ethical problems with respect to possible misuse of email consultation. Implementation problems occurred due to unclear policy on email consultation and the lack of training for email consultations. Caregivers found the applications too time consuming because these systems could not be integrated with their existing patient information system or medical records.

**Table 2:** Overview of caregiver problems (N = 198)

Quality Demand	Identified Caregiver Problems
User-friendliness (n = 101, 34.8%)	Navigation problems: Unclear navigation structure, hyperlinks lacking or useless Lack of feedback features Lack of documentation features Unclear answer procedures/formats
	Technical problems: Software bugs
Quality of care (n = 43, 37.9%)	Nonprofitability^*^ of email consultation: Requests from patients still required personal contact with a caregiver
	Concerns about a higher chance of interpretation difficulties: Carefulness with formulating answers to patient requests, such as being extremely careful when formulating the answer because of possible legal consequences
	Concerns about a higher chance of misuse: Requests from unknown patients through using the account of known patients
Implementation (n = 54, 27.3%)	Unclear regulations about email consultation: Lack of a transparent protocol for email consultation Unclear regulations about prerequisites for using email consultation Lack of quality inspection of email consultation applications Insufficient reimbursement for email consultation
	Lack of education and training: Underuse or misuse of applications because of lack of education
	Interoperability of systems: Applications could not be integrated with the existing patient information system or medical records
	Concerns about patient equity of access: Concerns about the risk of widening of the gap between those who have access to new technology and those who have been excluded

^*^Profitability: the degree to which the health service can be delivered in a quick, effective, and economical manner.

## Discussion

Patient and caregiver expectations did not correspond with their experiences with the use of the Internet-based applications for self-care. Patients thought that the applications would support them in solving their health problems, that they would guide them on a “problem-solving journey on the Internet” by consulting various interactive components that would enable them to make informed decisions about their health condition. Caregivers were more reserved about the applications because of medico-legal concerns about misuse. However, the applications failed to support self-care because eHealth is more than just a technological intervention. The design of the applications should include a way of thinking about how to deliver health care with the aid of technology [21]. The applications provide various interactive components disconnectedly, so users themselves have to find out which feature will be convenient and profitable for what purpose. In terms of diffusion of innovations [11], we know that only very motivated people will persist.

We aspired to determine user-centered criteria for Internet-based applications for self-care. We focused, therefore, on quality demands for interactive health communication applications as formulated in prior studies [2,11]: user-friendliness, quality of care, and implementation. Based on our results and prior studies, it can be concluded that technology should be simple and easy to use, in line with end users’ ways of thinking and behavior with respect to solving health problems via technology. Moreover, to develop or improve Internet-based applications for self-care, language and comprehensibility of information are important content criteria. Self-care support applications should match the vocabulary of the users and the language of the medical systems. This requires rethinking the presentation of information for self-control via the Internet. From the perspective of caregivers, the applications failed because of their inability to store medical data in the patient records already in use. The adoption of a new technology depends on the presence of an adequate infrastructure or other technologies that cluster with the innovation [11].

What health care functions can be delegated to Internet-based health care systems? We evaluated three applications with various components for self-care, such as symptom-driven question-and-answer systems, self-tests for preliminary evaluation of the urgency of a health complaint, and email consultation services for electronic patient-caregiver communication. Patients appreciated email communication more than the other components because they preferred convenient access to a high level of personalized health care. Digital triage was insufficiently geared to their expectations and was more medico-technology driven than user centered. The applications have multiple components for self-care to appeal a wide range of users, but without a thorough analysis of how people think and frame their problems, how they expect to be responsible for their own care and decisions, and what they need to support this self-care, the components might well result in an overload of information. People get lost on the Internet, so personal assistance is needed. In our opinion, we feel that the organization of patient-centered care expectation management is a prerequisite to delivering health care through technology.

Despite these shortcomings, we believe the applications have the potential to mature. The findings of our study are consistent with the results of previous studies [2,3,22-28]. For instance, the study by Car and Sheikh [24] presented key features for optimal email consultation, such as ease of adoption; combining new technology with existing ones; user-friendliness; easy to set up, manage, and use by doctors and patients; integration with existing medical records; and archiving and logging. These key features should therefore be addressed in the development of new Internet-based self-care applications. According to the Institute of Medicine [10], care needs to be customized according to patient needs and values, which we also found in our study. Problems related to quality of care resulted from patients’ inability to formulate their complaints as a health problem. The applications should be designed to solve this semantic problem by providing an adequate search engine and by avoiding the use of medical jargon. Moreover, the systems were incapable of delivering personalized and tailored health care, which seems one of the most important requirements for high-quality patient care. In order to improve the quality of care, applications should be designed to meet the most common types of need, but should also have the capability to respond to individual patient choices and preferences [10]. The Kerr et al study [2] identified quality criteria for Internet interventions for long-term conditions. The user-generated criteria relating to information content, presentation of information, language, and interactivity (tailored and personalized advice, question-and-answer functionality) correspond with the findings of our study in the sense that the absence of these criteria impeded self-care.

This correspondence in study findings illustrates that Internet-based technology in health care is evolving throughout the world and that it encompasses comparable quality demands. Although the impact of Internet-based technology may not be fully clear until diffusion becomes widespread, explorative studies such as this one can give insight into the requirements necessary for widespread use in the future.

The use of scenario-based tests combined with in-depth interviews proved to be a powerful method for describing and identifying user problems and for supporting the re-design processes of the Internet-based applications for self-care. From prior studies [4,29], we know that such a qualitative approach provides reliable and meaningful data for developing and implementing Internet-based technology for supporting self-care. Moreover, the use of the scenario-based tests provided patients and caregivers with the opportunity to learn about the functionality of the applications and how to use them more efficiently, and it gave them more confidence in the utility of the Internet-based technology.

Notwithstanding the relatively small size of our sample, which limits the generalizability of our results, we now have more insight into the requirements for successful Internet-based technology for supporting self-care. The aforementioned criteria on user-friendliness, quality of care, and implementation of the technology are key elements in creating an efficient and effective Internet consultation process. To foster widespread use of Internet-based technology, like electronic patient-caregiver communication and self-assessment via the Internet, the needs of end users should be the starting point for the development of such applications [29-31]. In order to prevent the risk of providing inaccurate or inadequate advice, self-assessment tools that are neither efficient nor effective should not be part of eHealth services. The most powerful application for self-care is email consultation, combined with a suitable triage mechanism to empower patients’ self-awareness.

There will be ongoing demand for evaluation of eHealth services. Future studies should focus on the possibilities of self-care via Web-based triage systems combined with email communication to create awareness of illness and to make timely care possible and feasible. These systems should be interoperable with electronic health records and tailored to particular usage (ie, users with comparable disease profiles).

## Multimedia Appendix

Scenarios used in the study
