# Supplementary material for: Evaluation of Internet-Based Technology for Supporting Self-Care: Problems Encountered by Patients and Caregivers When Using Self-Care Applications
Source: J Med Internet Res. 2008 May 15;10(2):e13. doi: 10.2196/jmir.957 (PMC2483926; doi:10.2196/jmir.957)
Supplement: Supplementary file 1 [file jmir_v10i2e13_app1.pdf]

Nijland N, van Gemert-Pijnen JE, Boer H, Steehouder MF, Seydel ER
Evaluation of Internet-Based Technology for Supporting Self-Care: Problems Encountered by Patients and Caregivers When Using Self-Care Applications
J Med Internet Res 2008;10(2):e13
<URL: http://www.jmir.org/2008/2/e13/>

Multimedia Appendix: Scenarios used in the study

Scenario cough: "I have a cold, and for three days I have had a tickling cough. I haven't got a fever, a headache or earache. I also don't cough up phlegm. I'm feeling a bit ill, but I can still work. I'm not using any medication at the moment. I think I'm all right, but I need to be sure, because the day after tomorrow I'm going on holiday to Thailand. Therefore, I need to know what precautions I need to take, like cough medicine."

Scenario tiredness: "Lately, I have had some concentration problems at my work. I work as an engineer at an advice centre. I don't sleep very well, and I worry a lot without reason. My family life is all right; I haven't got problems with my wife or children. My work pleases me, too, but sometimes I get really quick annoyed by a colleague. At times, I let rip at a colleague unintentionally. First, I thought I was having a burn-out, or rather: my colleagues thought I was. I don't think I have a burn-out. I like my job. The workload is not so bad either. Still, I'm worried. I'm so listless. I would like to know what it could be. I'd rather not go to a psychologist who would immediately label my problem as a burn-out."

Scenario excessive sweating: "I often have trouble with excessive sweating. Especially during official events, I get really irritated. I have tried several deodorants, but they don't seem to work. I noticed that I don't look forward to receptions and presentations, which

are part of my work as a salesman. It's making me insecure. Is there some kind of cure against sweating? Will this be covered by my insurance?"

Scenario relational problems: "Recently, I ended my relationship. We had plans to live together, but eventually we decided not to. Right now, I feel lonely, which gives me doubts about my decision to break up. My ex-boyfriend has doubts, too, but he doesn't seem to be so bothered. Unlike me, he is getting on with his life. I remain doubtful for we have known each other since high school. Would relationship therapy be useful for us?"

Scenario sexually transmittable disease: "Recently, I've had a boyfriend. I'm a little bit concerned, because my boyfriend had a girlfriend who had a sexually transmittable disease (she once told me). I'm afraid to ask my boyfriend if he had sex with her. Therefore, I would like to know how I can recognize a sexually transmittable disease."

Scenario vaccination: "This year, I'm going with a party of tourists to Peru during the winter. I am considering going to the Amazon region and I heard that malaria exists in this area. The thing is that I'm rather sensitive to medication (I get a rash, etc.). I'm only going there for three days. Therefore, I would like to know whether it is absolutely necessary to take medication. If necessary, which combination of malaria medication should I take, and which medication gives the least side effects? I would also like to know when (at what moment of the day) I should take the medication. Moreover, am I still insured if I decide not to take medication?"
